# Whole blood transcriptomic analysis reveals PLSCR4 as a potential marker for vaso-occlusive crises in sickle cell disease

**DOI:** 10.1038/s41598-021-01702-8

**Published:** 2021-11-12

**Authors:** Hawra Abdulwahab, Muna Aljishi, Ameera Sultan, Ghada Al-Kafaji, Kannan Sridharan, Moiz Bakhiet, Safa Taha

**Affiliations:** 1 Department of Molecular Medicine, College of Medicine and Medical Sciences, Princess Al Jawhara Center for Molecular Medicine, Genetics and Inherited Diseases, Arabian Gulf University, Manama, Kingdom of Bahrain; 2 Department of Pharmacology and Therapeutics, College of Medicine and Medical Sciences, Arabian Gulf University, Manama, Kingdom of Bahrain

**Keywords:** Gene expression, Sickle cell disease

## Abstract

Sickle cell disease, a common genetic blood disorder, results from a point mutation in the β-globin gene affecting the configuration of hemoglobin, predisposing to painful vaso-occlusive crisis (VOC) and multi-organ dysfunctions. There is a huge variation in the phenotypic expressions of SCD and VOC owing to genetic and environmental factors. This study aimed to characterize the whole blood gene expression profile using Microarray technology in Bahraini patients with SCD determining the differentially expressed genes in steady-state (n = 10) and during VOC (n = 10) in comparison to healthy controls (n = 8). Additionally, the study intended to identify potential genetic marker associated with hemolysis. The analysis identified 2073 and 3363 genes that were dysregulated during steady-state and VOC, respectively, compared to healthy controls. Moreover, 1078 genes were differentially expressed during VOC compared to steady state. The PLSCR4 gene was almost 6-fold up-regulated in microarray, 4-fold in polymerase chain reaction, and a mean protein concentration of 0.856 ng/ml was observed in enzyme-linked immunosorbent assay during VOC compared to steady-state (0.238 ng/ml) (*p* < 0.01). Amongst these genes, PLSCR4 is involved in erythrocyte membrane deformity thus, predisposing to hemolysis, adhesion, and thrombosis. In conclusion, PLSCR4 may serve as a potential biomarker for VOC and future large-scale validation are recommended.

## Introduction

Sickle cell disease (SCD) is a group of inherited blood disorders caused by a mutation in the hemoglobin gene that results in the formation of hemoglobin S (HbS) and leads to hemolytic anemia, painful attacks and multiple organ dysfunctions^1,2^. Varied presenting symptoms (acute/chronic/with complications) are observed in SCD^3^. The most common acute presentation include painful vaso-occlusive crises (VOC) requiring urgent care in emergency department or hospitalization, thus resulting in a huge burden to the patient/s and the healthcare providers^4,5^. Poor health-related quality of life was observed in Bahraini patients with SCD due to due to frequent admissions to the hospital^6^.

Moreover, although SCD is a monogenetic disorder, it is clinically heterogeneous^7^. There is a huge variation in the phenotypic expression due to multiple factors influencing the clinical manifestations and disease outcomes^8^. The major modulators of SCD phenotype are the concentrations of hemoglobin F (HbF) and the coinheritance of α-thalassemia that affects the HbS polymerization, ameliorating the hematological features and disease severity^9–11^. Additionally, several genetic polymorphisms affect the SCD phenotype^12,13^. Even then, gap remains regarding attributing the factors related to differences in the phenotypic expression of SCD and VOC^11^. Moreover, the gene–gene and gene-environment interactions can influence the disease phenotype due to which extrapolation from western studies may not be biologically plausible due to variations in the genetic expressions^12,13^.

This study aimed to compare SCD patients using gene expression analysis by microarray to improve our knowledge of the disease pathophysiology and to detect genetic markers associated with the VOC. Identifying such markers may aid in the development of targeted therapy to treat SCD and prevent VOC.

## Results

### Characteristic of participants

Twenty sickle cell disease patients and their characteristics are listed in Table 1. Majority of the study participants were male with a mean age of 33 years. The frequency of VOC among steady-state group was 9.7 ± 6.16 per year in which 34% needed hospital admission, while the VOC frequency was 8.3 ± 5.38 per year in the VOC group from which nearly half of the attacks (44.6%) ended up with hospital admission.

**Table 1:** Baseline characteristics of the study participants with SCD (n = 20).

Parameters	Steady-State	VOC	*p-*values
Gender [n (%)]			
Male	9 (90)	9 (90)	1
Female	1 (10)	1 (10)	1
Age Mean in years ± SD	33 ± 10.82	34.9 ± 9.3	0.68
No. of VOC per year ± SD	9.7 ± 6.16	8.3 ± 5.38	0.59
White Blood Cell counts Mean in × 10^9^/L ± SD	5.4 ± 2.95	6.05 ± 3.91	0.68
Red Blood Cell counts Mean in × 10^12^/L ± SD	4.89 ± 0.94	4.07 ± 0.95	0.07
Mean corpuscular volume Mean in fL ± SD	73.86 ± 10.48	82.01 ± 12.58	0.13
Mean corpuscular hemoglobin Mean in pg ± SD	23.53 ± 4.55	26.6 ± 5.49	0.19
Hemoglobin Mean in g/dL ± SD	11.17 ± 1.14	10.48 ± 1.51	0.27
Hematocrit Mean in % ± SD	35.33 ± 3.82	32.49 ± 4.58	0.15
Platelet Mean in × 10^9^/L ± SD	309.19 ± 205.84	201.2 ± 118.04	0.17
Retics Mean in % ± SD	5.5 ± 2.23	6.96 ± 4.7	0.39
Hemoglobin F Mean in % ± SD	13.88 ± 8.3	18.26 ± 6.02	0.2
Hemoglobin S Mean in % ± SD	79.81 ± 7.97	76.25 ± 5.41	0.26
Bilirubin mean in µmol/L ± SD			
Direct	10.1 ± 3.45	18.3 ± 9.81	0.03
Indirect	20.7 ± 10.76	35.02 ± 17.35	0.04
Lactate dehydrogenase Mean in U/L ± SD	314.2 ± 117.2	572.4 ± 764	0.32

Moreover, laboratory assessments of the steady-state group revealed that their parameters were within the normal range: white blood cell (WBC), red blood cell (RBC) and platelet within a normal range 5.4 ± 2.95 × 109/L, 4.89 ± 0.94 × 1012/L and 309.19 ± 205.84 × 109/L, respectively, with low indices (mean corpuscular volume (MCV) 73.86 ± 10.48 fL and mean corpuscular hemoglobin (MCH) 23.53 ± 4.55 pg). The hemoglobin level was low 11.17 ± 1.14 g/dL with hematocrit of 35.33 ± 3.82%. On the other hand, in the VOC group, the total count of WBC 6.05 ± 3.91 × 109/L, RBC 4.07 ± 0.95 × 1012/L and platelet 201.2 ± 118.04 × 109/L with normal indices MCV 82.01 ± 12.58 fL and MCH 26.6 ± 5.49 pg. The hemoglobin level was low 10.48 ± 1.51 g/dL with the hematocrit of 32.49 ± 4.58%.

The statistical analysis showed no significant differences between both groups in baseline characteristics (*p*-value > 0.05). However, there was a statistically significant difference among the two groups (steady-state, VOC) in the level of hyperbilirubinemia in form of direct bilirubin (10.1 ± 3.45, 20.7 ± 10.76 in µmol/L, *p* = 0.03) and indirect bilirubin (20.7 ± 10.76, 35.02 ± 17.35 in µmol/L, *p* = 0.04).

### Determination of the differentially expressed genes

**Figure 1:**
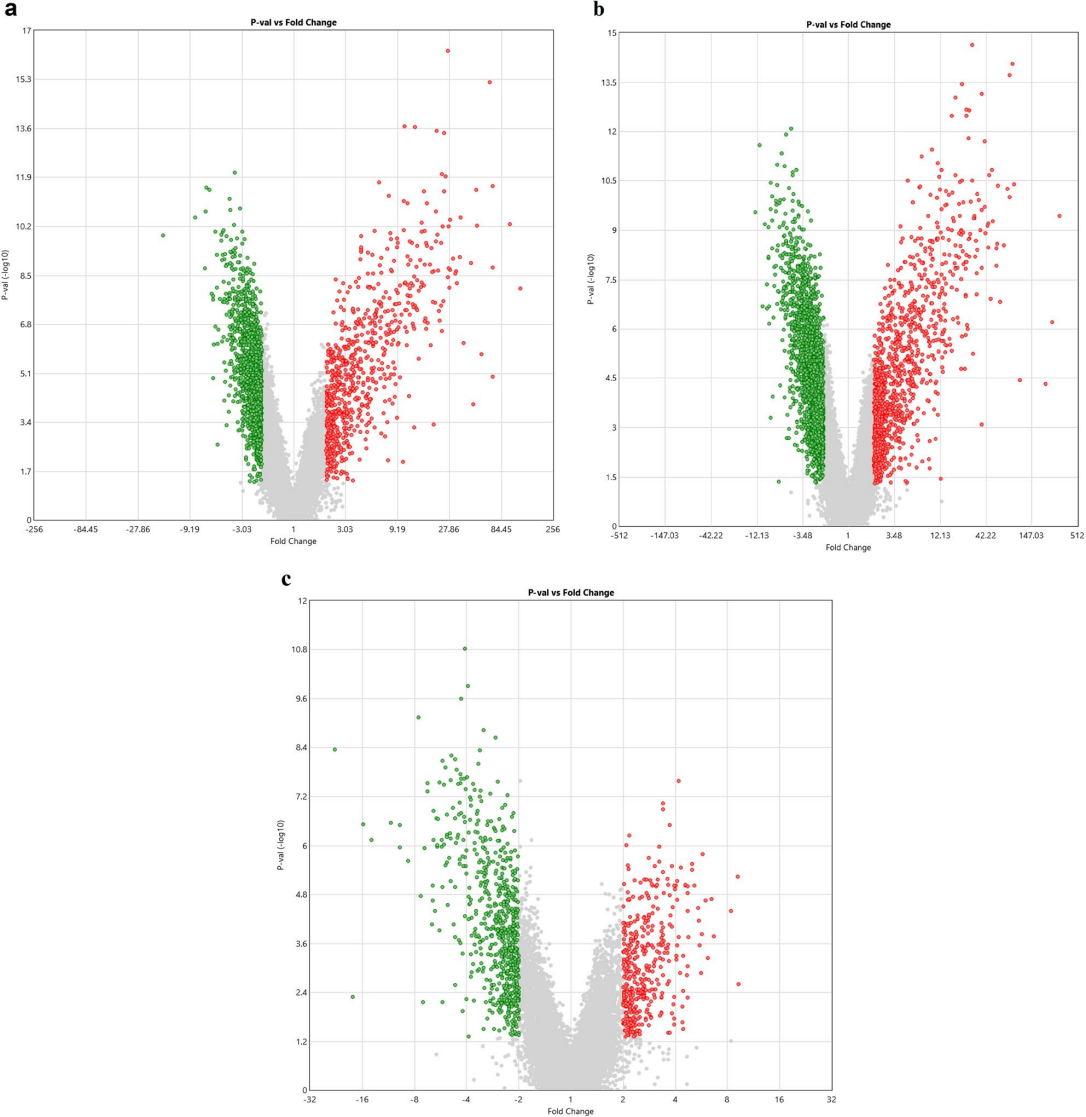
Volcano plots of the differentially regulated genes at a *p-value* of < 0.05 and a fold change of < − 2 or > 2. (**a**) 2073 differentially regulated genes in SCD patients in steady-state compared to healthy controls. (**b**) 3363 differentially regulated genes in SCD patients in VOC compared to healthy controls. (**c**) 1078 differentially regulated genes in SCD patients in VOC compared to SCD patients in steady-state.

A total of 2073 genes were dysregulated in which 736 genes were up-regulated (*p* < 0.05) with a fold change of > 2, and 1337 genes were down-regulated (*p* < 0.05) with a fold change of < − 2 in SCD patients in steady-state compared to healthy controls (Fig. 1a). Whereas, in SCD patients in VOC compared to healthy controls, 3363 genes were differentially regulated including 1080 genes were up-regulated (*p* < 0.05) with a fold change of > 2 and 2283 genes were down-regulated (*p* < 0.05) with a fold change of < − 2 (Fig. 1b). In addition, 1078 genes were differentially expressed including 410 up-regulated genes and 668 down-regulated genes in SCD patients in VOC compared to steady-state (*p* < 0.05) with a fold change of > 2 and < − 2, respectively (Fig. 1c).

Furthermore, at a *p*-value of < 0.001 and a fold change of > 4 a total of 292 genes were up-regulated in SCD patients in steady-state compared to healthy controls, while in SCD patients in VOC compared to healthy controls 428 genes were up-regulated, Table 2. Additionally, 31 genes were up-regulated in SCD patients in VOC compared to steady-state.

**Table 2:** Top ten up-regulated genes at *p*-values of < 0.001 and a fold change of > 4.

ID	Gene Symbol	Chromo-some	Group	*p-*values	Fold Change
**A. SCD patients in steady-state compared to healthy controls**					
TC0100015815.hg.1	THEM5	chr1	Coding	9.26E−09	126.94
TC0400012018.hg.1	GYPB	chr4	Multiple_Complex	5.24E−11	101.13
TC1000008399.hg.1	IFIT1B	chr10	Coding	1.70E−09	70.57
TC0100011325.hg.1	TMCC2	chr1	Multiple_Complex	2.54E−12	70.46
TC0Y00006730.hg.1	EIF1AY	chrY	Multiple_Complex	1.04E−05	69.74
TC0700009232.hg.1	BPGM	chr7	Coding	6.30E−16	66.21
TC1400008056.hg.1	IFI27	chr14	Multiple_Complex	1.70E−06	54.87
TC0100017118.hg.1	YOD1	chr1	Multiple_Complex	6.08E−11	50.36
TC1200008726.hg.1	TCP11L2	chr12	Multiple_Complex	3.47E−12	49.56
TC0600007006.hg.1	RNF182	chr6	Coding	9.59E−05	45.94
**B. SCD patients in VOC compared to healthy controls**					
TC1000008399.hg.1	IFIT1B	chr10	Coding	3.65E−10	313.47
TC1400008056.hg.1	IFI27	chr14	Multiple_Complex	6.24E−07	255.9
TC0Y00006730.hg.1	EIF1AY	chrY	Multiple_Complex	4.66E−05	211.92
TC0100013223.hg.1	RAP1GAP	chr1	Multiple_Complex	3.56E−05	105.06
TC0900010959.hg.1	HEMGN	chr9	Multiple_Complex	3.99E−11	90.78
TC0700009232.hg.1	BPGM	chr7	Coding	8.49E−15	86.56
TC1800006889.hg.1	RIOK3	chr18	Multiple_Complex	1.88E−14	80.18
TC0100017118.hg.1	YOD1	chr1	Multiple_Complex	9.70E−11	79.84
TC1300008424.hg.1	USP12	chr13	Multiple_Complex	5.42E−11	75.5
TC0400012018.hg.1	GYPB	chr4	Multiple_Complex	2.86E−09	68.5

**Figure 2:**
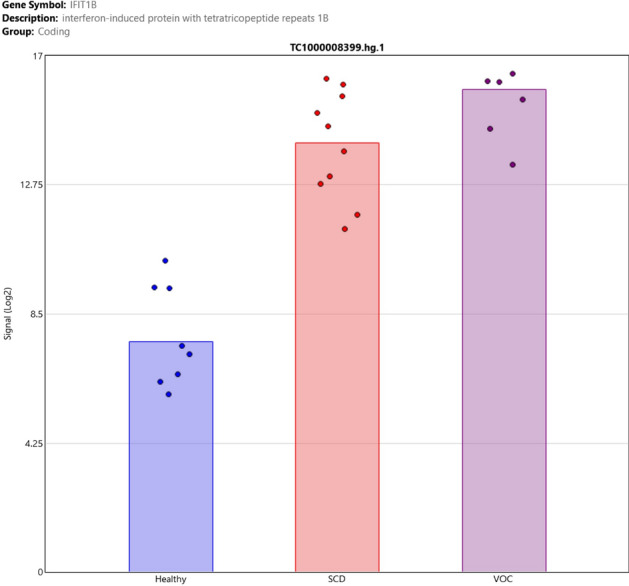
Sample signals of IFIT1B gene. Sample signals showing up-regulation in SCD patients in steady-state and VOC compared to healthy controls with a false discovery rate of 5.41 × 10^–8^ and a fold change of 70.57 and 313.47, respectively.

Moreover, the IFIT1B (Interferon Induced Protein with Tetratricopeptide Repeats 1B) gene shows the highest fold change among the differentially regulated genes as it was up-regulated in SCD patients in steady-state compared to healthy controls with a fold change of 70.57 (*p* = 1.7e−9), as well as in SCD patients in VOC compared to healthy controls at a fold change of 313.47 (*p* = 3.65e−10) (Fig. 2).

### Potential genetic marker for vaso-occlusive crisis

To identify a potential genetic marker for VOC, the thirty-one up-regulated genes were assessed to define the “True up-regulation” against those genes that were up-regulated at a *P*-value of < 0.001 with 4-fold change in SCD patients in VOC compared to SCD patients in steady-state and in SCD patients in VOC compared to healthy controls, but not more than 2-folds down-regulated in SCD patients in steady-state compared to healthy controls at a *P*-value of < 0.05. A total of 13 up-regulated genes remained, Table 3.

**Table 3:** Differentially regulated genes in SCD patients in VOC compared to SCD patients in steady-state.

ID	Gene symbol	Chromo-some	*p-*values	Fold change		
				VOC vs. Steady-state	VOC vs. Healthy	Steady-state vs. Healthy
TC2000007117.hg.1	ASXL1	chr20	5.81E−06	9.13	13.2	2
TC0100018440.hg.1	STIL	chr1	3.98E−05	8.34	10.62	1.93
TC0800008726.hg.1	TBC1D31	chr8	2.05E−05	6.49	10.21	1.82
TC0300012720.hg.1	PLSCR4	chr3	1.63E−06	5.76	9.38	1.79
TC1500008023.hg.1	ZFAND6	chr15	0.0001	5.71	9.07	1.69
TC0500008830.hg.1	UBE2D2	chr5	9.37E−06	5.12	8.05	1.58
TC1100009819.hg.1	NAP1L4	chr11	2.86E−06	5.02	7.87	1.56
TC1200007906.hg.1	XRCC6BP1	chr12	0.0002	4.74	7.54	1.5
TC1400009108.hg.1	POLE2	chr14	0.0009	4.73	7.28	1.44
TC2200009278.hg.1	RBX1	chr22	9.82E−06	4.7	6.65	1.41
TC1200007137.hg.1	FGFR1OP2	chr12	1.43E−05	4.69	6.57	1.4
TC0700012044.hg.1	FIS1	chr7	3.48E−06	4.32	5.45	1.39
TC0600009080.hg.1	CEP57L1	chr6	2.62E−08	4.18	5.39	1.34

**Figure 3:**
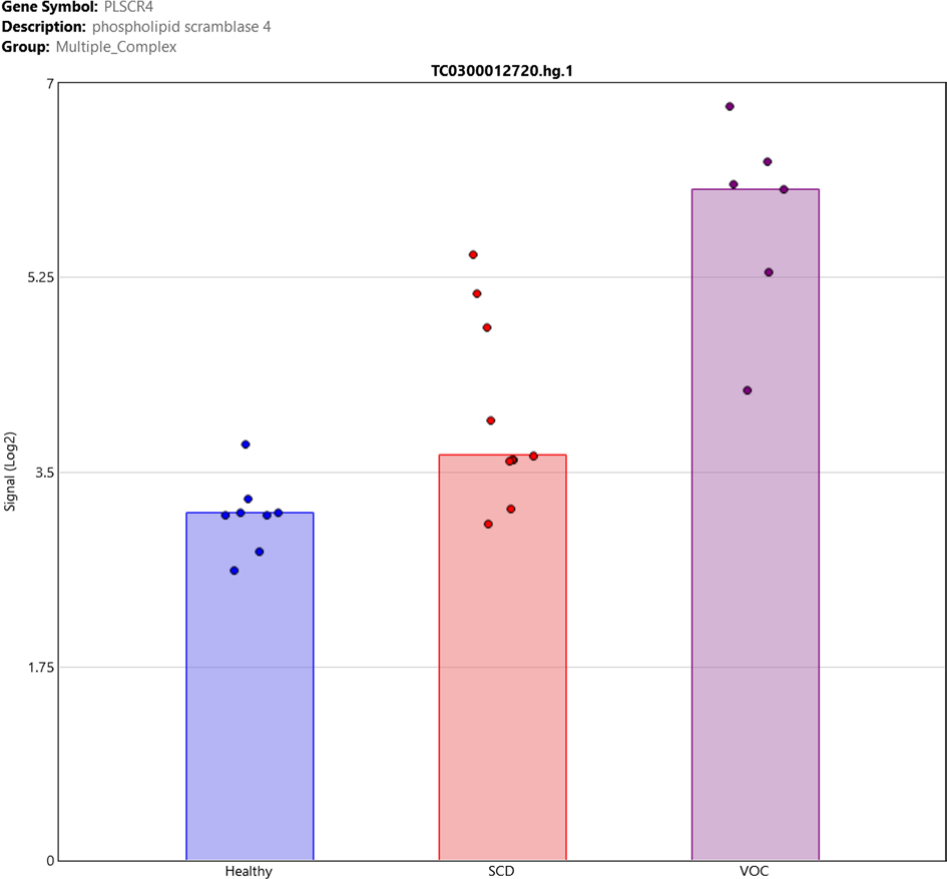
Sample signals of PLSCR4 gene. Sample signals of PLSCR4 gene showing almost 6-folds up-regulation in SCD patients in VOC compared to SCD patients in steady-state.

Following the data mining, an up-regulated gene PLSCR4 (Phospholipid Scramblase 4) was selected to be assessed by qRT-PCR and ELISA and further studied in association with hemolysis and inflammation. The PLSCR4 gene showed almost 6-folds up-regulation in SCD patients in VOC compared to SCD patient in steady-state (*p* = 1.63e−6) (Fig. 3).

### Validation of PLSCR4 through qRT-PCR and ELISA

**Figure 4:**
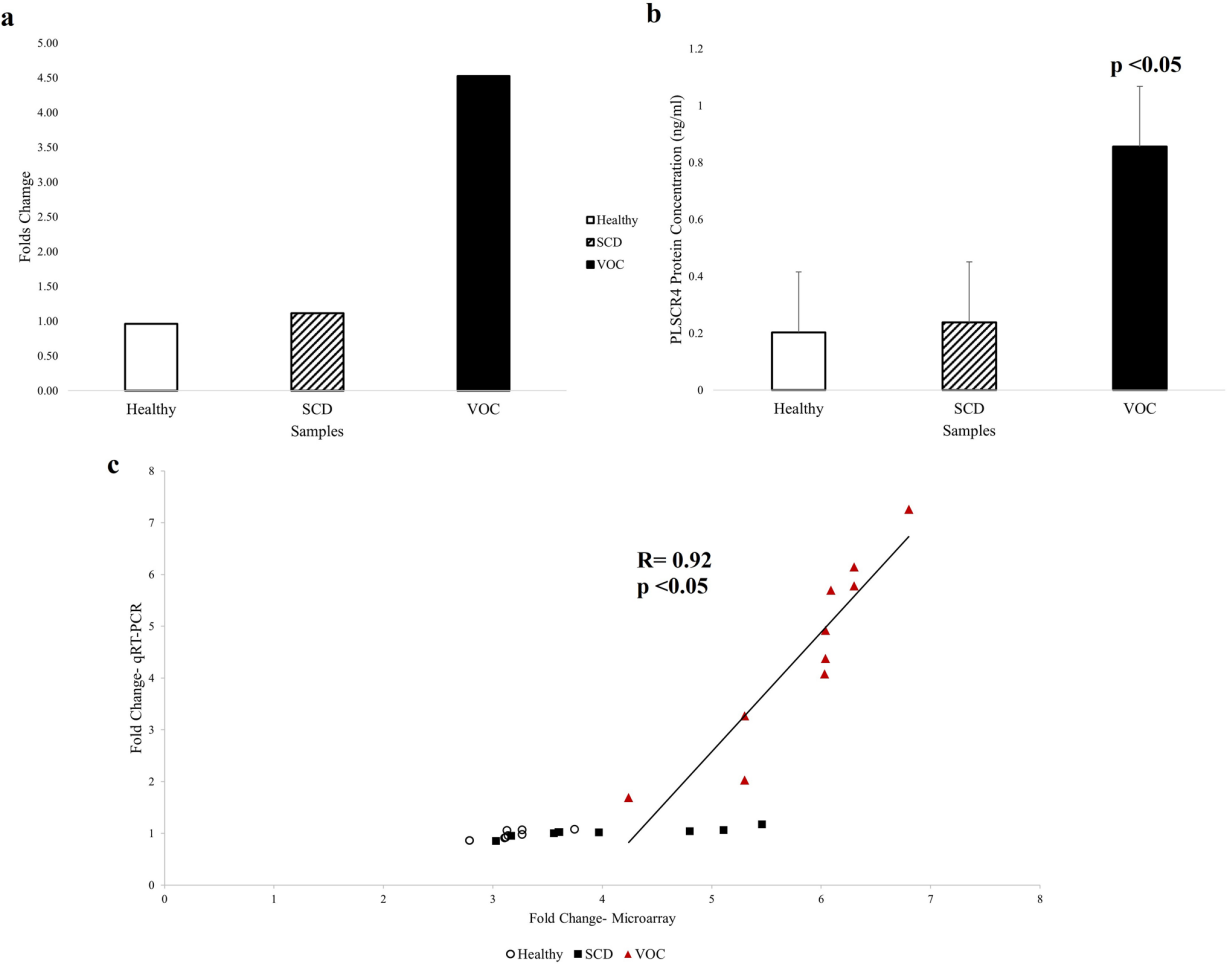
PLSCR4 gene expression level and protein concentration. (**a**) Average folds change of PLSCR4 gene through real-time polymerase chain reaction (qRT-PCR) showing 4-folds up-regulation in SCD patients in VOC compared to SCD patients in steady-state at a *p*-value of 0.00017. (**b**) The average PLSCR4 protein concentration was increased in SCD patients in VOC compared to SCD patients in steady-state at a *p*-value of 9.072 × 10^–6^. (**c**) Correlation of fold change of PLSCR4 gene expression measured by microarray and qRT-PCR. X-axis represents log2-fold change determined by microarrays; y-axis represents log2-fold change determined by qRT-PCR.

The analysis of PLSCR4 gene expression by qRT-PCR showed statistically significant up-regulation in SCD patients in VOC compared to SCD patient in steady-state with a 4-fold changes (*p* = 0.00017) (Fig. 4a,c). Moreover, the PLSCR4 gene expression was confirmed by measuring the protein concentration using ELISA. The assay showed a significant increase in PLSCR4 expression in SCD patients in VOC (0.856 ng/ml) compared to SCD patient in steady-state (0.238 ng/ml) (*p* = 9.072e−6) (Fig. 4b).

## Discussion

In SCD, numerous biomarkers were identified to be involved in the disease pathophysiology^14^ but their clinical utility was questioned owing to influence of several factors are contributing to inflammation, oxidative stress, adhesion and coagulation^15,16^. Few studies were conducted in detecting the transcriptomic biomarkers of SCD and their results are encouraging; however, these studies were carried out in West African population^17,18^. To the best of our knowledge, this is the first study to explore the use of microarray technology in determining the whole blood gene expression profiles in Arabs with SCD.

Recently, a gene expression meta-analysis combined with a genome-wide association study data analysis identified that major pathways involved in SCD include innate immunity, hemostasis, response to stress, hemopoiesis, heme biosynthesis and apoptosis^19^. Several up-regulated genes identified in the previous transcriptomic studies were also found to be up-regulated in our study, notably the genes related to erythrocyte development and innate immune system. Also, the meta-analysis discovered some of protein coding genes that were not previously studied in relation to SCD including RUNDC3A, TMCC2, OSBP2 and IFI27^20^ similar to the present study.

However, characteristically, only in our study population, the IFIT1B gene was the highest up-regulated gene in SCD patients in both states with a higher fold change in patients with VOC. This gene was not previously studied in relation to SCD. According to the GeneCards database, the IFIT1B is a protein coding gene, which has a part in the Toll-like Receptor signaling pathway^21^ and belongs to the IFIT family which exhibits an antiviral activity^22^. Moreover, the GeneHancer revealed a relation between IFIT1B gene and phenotype SNPs in leukocyte count, C-reactive protein measurement and myocardial infarction^23^. Nevertheless, IFIT1B gene is uncharacterized yet and its function and expression need to be identified^22^.

In our study, the PLSCR4 was significantly up-regulated in SCD patients in VOC compared to SCD patient in steady-state and this was further confirmed by qRT-PCR and ELISA. These results suggest that PLSCR4 gene may serve as a potential genetic marker for the development of VOC in SDC patients.

The PLSCR4 was not previously identified in studies investigated gene expression in SCD patients and was not previously assessed for its role at transcriptional level in SCD patients.

PLSCR4 is a protein coding gene located on chromosome 3q24 and belongs to the scramblase family^21^. The gene function facilitates fast bidirectional transbilayer transportation of phospholipids including PS which triggered by Ca2 + binding and independent of ATP resulting on loss of the normal asymmetry of plasma membrane^24,25^. Additionally, it has a crucial role in fibrin clot formation initiation, mast cells activation, detection and removal of damaged cells by the reticuloendothelial system^26–30^.

A study on murine models of SCD found that majority of sickle mice RBCs exhibit phospholipid scramblase that was linked to phosphatidylserine (PS) externalization, in addition, the results reveal a rapid peripheral destruction of RBCs which was strongly correlated with the degree of scramblase activation suggesting that scramblase activity and PS exposure serve as markers of hemolysis^31^. Moreover, PS-exposing cells act as a target for interaction with proteins and enzymes in plasma such as secretory phospholipase A2 which is a potent lipid mediator in inflammation as it hydrolyzes the lipids in PS-exposing RBCs producing lysophospholipids and free fatty acids that affects vascular integrity contributing to vascular damage, as well as secretory phospholipase A2 was identified to be involved in the development of acute chest syndrome^32,33^.

Likewise, scramblase function has a major role in SCD pathogenesis especially in initiating VOC as it facilitates cell adhesion, aggregation, increasing hemolysis, vascular dysfunction, promoting coagulopathy and inflammation which is the hallmark of microvascular occlusion, yet its mechanism in SCD is still not fully explored and need to be further studied^32^. Furthermore, in a study done by Francis et al. to characterize the function of human phospholipid scramblase 4 (hPLSCR4) which is an isoform of the scramblase family through cloning a recombinant hPLSCR4, it confirms that hPLSCR4 is a Ca2 + -binding protein and identified a point mutation (Asp290 → Ala) resulting in almost 50% reduction in scramblase activity in the presence of Ca2 + ^34^.

In addition, Kenneth Dobie invented a patent antisense oligonucleotide (US 10/673,523) targeting nucleic acids encoding phospholipid scramblase 4 and modulating its expression for treatment of diseases such as Scott syndrome^35^. Such a discovery will open the path for treating diseases associated with increased expression of PLSCR4. And based on our study finding this may help as a targeted therapy for SCD patient to reduce the risk of developing VOC, chronic anemia and other disease complications.

In conclusion, our study characterized the transcriptomic signature in Bahraini SCD patients with highlights on the role of hemolysis and inflammation in disease state. Analyzing the transcriptional changes in SCD during two status (steady-state and VOC) resulted in the discovery of new genes to be associated with SCD for the first time and were significantly differentially expressed. Amongst these genes, PLSCR4 is involved in causing RBC membrane deformity thus, predisposing to hemolysis, adhesion, and thrombosis. Further validation in a larger sample size is recommended and its pathway needed to be further studied in relation to the disease pathogenesis as it may serve as a potential genetic biomarkers and aids in the discovery of novel therapeutic target. Finally, the study yielded a transcriptomic database of SCD patients from Arab’s ethnicity that may help future studies in further understanding the disease heterogeneity and facilitating the development of personalized medicine and targeted treatment in managing SCD patients.

## Methods

### Study population

Twenty Bahraini patients with SCD (10 at steady state and 10 with VOC) and eight healthy participants from Salmaniya Medical Complex were recruited in this cross-sectional study during September 2019. Approval from the Research and Ethics Committee of the Arabian Gulf University, and the Research Technical Support Team of the Ministry of Health, Kingdome of Bahrain were obtained. Written informed written consents were obtained from each study participant. All experiments and methods were performed in accordance with relevant guidelines and regulations.

The healthy volunteers were confirmed to have Hemoglobin AA genotype by High Performance Liquid Chromatography (HPLC), while all patients with SCD were confirmed to have HbSS genotype. SCD patients were divided into two groups of ten participants each: SCD patients in steady state defined as participants without any history of VOC that required neither evaluation in an emergency department nor hospital admission 12 weeks prior to the study enrollment; and SCD patients during VOC defined as participants with a history of acute, severe pain at the time of enrollment (self-rated score of ≥ 7 out of 10 on a Numerical Rating Scale (NRS))^36,37^. All SCD patients were not under treatment with hydroxyurea.

### Sample collection

For all subjects, blood samples were collected in two separate tubes. For VOC group, the samples were collected within the first 48 h of the crisis. First, 5 ml of venous blood were collected in serum-separating tube and kept for 30 min in room temperature for clot formation and then centrifuged at 3500 rpm for 15 min. The separated serum was stored at − 80 °C until the analysis. In the second container, 2.5 ml of venous blood were collected in PAXgene® Blood RNA Tube (PreAnalytiX GmbH, Hombrechtikon, Switzerland) for immediate stabilization of intracellular RNA and was kept for minimum 2 h at room temperature to allow for complete lysis of blood cells, and then stored at 4 °C until the analysis that was carried out within 3 days.

### RNA extraction and gene expression analysis

RNA was extracted from whole blood samples using PAXgene® Blood RNA kit (PreAnalytiX GmbH, Hombrechtikon, Switzerland) following the manufacturer’s instructions. The quantity and purity of RNA samples were determined using the NanoDrop 1000 Spectrophometer (Thermo Fisher Scientific, Inc., Waltham, MA, USA) and the acceptable RNA purity of A260/A280 was 1.8–2.2. The RNA integrity was assessed using 1.2% agarose gel electrophoresis. All RNA samples were stored at − 80 °C until further analysis.

The assessment of gene expression was carried out using Affymetrix ClariomTM S Assays for human and GeneChip™ WT PLUS Hybridization, Wash and Stain Kit (Applied Biosystems™, California, USA) according to the manufacture’s protocol. In brief, reverse transcription 100 ng of total RNA of each sample was converted to double-stranded cDNA using the T7 promoter sequence primer. Followed by synthesis and amplification of cRNA by an in vitro transcription of the second-stranded cDNA using T7 RNA polymerase. Then through reverse transcription of cRNA, the second cycle of single-stranded cDNA was synthesized, which contains dUTP. After hydrolyzing the RNA, 5.5 μg of purified single-stranded cDNA was fragmented using uracil-DNA glycosylase and apurinic/apyrimidinic endonuclease 1. Next, by terminal deoxynucleotidyl transferase the fragmented cDNA was labeled with DNA Labeling Reagent which binds to biotin. The fragmented and biotin-labeled single-stranded cDNA samples were hybridized to GeneChip™ WT PLUS for sixteen hours in Affymetrix GeneChip® Hybridization Oven 645. Followed by washing and staining using the Affymetrix GeneChip® Fluidics Station 450 and Affymetrix® GeneChip® Command Console™ (AGCC) software. Finally, the arrays were scanned using Affymetrix GeneChip® Scanner 3000 7G.

### Quantitative real-time polymerase chain reaction

Real-time polymerase chain reaction (PCR) was performed to measure the expression of Human Phospholipid scramblase 4 (PLSCR4) gene and normalized to GAPDH as a housekeeping gene. The reaction mixture for SYBR Green assay contained 2 × SYBR™ Select Master Mix (Applied Biosystems™, California, USA), 10 pmol of each forward and reverse primers (metabion international AG, Planegg, Germany) and 50 ng of cDNA.

The sequences of the primers for PLSCR4 and GAPDH were as follows: PLSCR4 forward primer, 5′-CATGGGTCTCTGGCGTTT CT-3′, and PLSCR4 reverse primer 5′-AGTTTGTAC GGTGCCCT-3′; GAPDH forward primer 5′-TCCCTGAGCTGAACGGGAAG-3′, and GAPDH reverse primer 5′- GGAGTGGGTGTCGCTGT -3′.

The reaction was carried out in 20 µL capillaries and incubated in Light Cycler® 2.0 (Roche). The used LightCycler run protocol was as the follows: Denaturation program at 95 °C for 10 min, amplification and quantification program repeated 45 times at 95 °C for 10 s, 60 °C for 30 s and 72 °C for 30 s, and finally a cooling step at 40 °C for 30 s. The accumulation of PCR products during each cycle was determined by observing the rise in fluorescence of DNA-binding SYBR Green. Afterwards, the crossing point of each sample was detected and normalized to the expression of housekeeping gene. Then the fold change of expression was calculated using the 2^-ΔΔCt method.

### Enzyme-linked immunosorbent assay

The protein produced by PLSCR4 gene was measured by Enzyme-Linked Immunosorbent Assay (ELISA) (MyBioSource, California, USA) according to the manufacturer’s instructions. The assay was performed by assigning duplicated wells for all standards and samples on plates, then 100 µL per well of standard or serum sample were pipetted into the assigned well. After incubation and washing, a 100 µL of Biotin-Conjugate was added, incubated, and washed. After that, a 100 µL of Streptavidin-HRP was added, incubated, and washed. Then, a 100 µL of Substrate Solution was pipetted to each well and incubated at 37 °C for 15–20 min. After getting the desired blue color intensity, the reaction was terminated by adding a 50 μL of Stop Solution to each well. Immediately, the optical density (OD) at 450 nm was measured for each well using Synergy™ HTX Multi-Mode Microplate Reader (BioTek®) (BioTek Instruments, Inc., Winooski, VT, USA) and analyzed by Gen5 2.07.17.

### Statistical analysis

The demographic data were analysed for their differences in SCD patients in steady state and during VOC. The values of continuous data were analysed by Student’s two-sided unpaired t-test and presented in mean ± standard deviation (SD). The categorical variables were presented in numbers (percentage) and were analysed using Fisher’s exact probability test. *p*-value of < 0.05 was considered significant.

The Transcriptome Analysis Console (TAC) software version 4.0.0.25 by Thermo Fisher Scientific were used to define the differential expression profile within the different groups, performs statistical analysis and provides a list of differentially expressed genes. Genes with a fold change of > 2 or < − 2 and with a t-test or ANOVA *P*‐value of < 0.05 were considered significantly altered between the conditions of each group.

The Light Cycler Software version 4.1.1.21 was used for the analysis of qRT-PCR results by identifying the crossing points for the target and the reference gene in each sample. Average of crossing points for each target gene was calculated in relative to the housekeeping gene GAPDH in all the groups using relative Mono-Color Relative Quantification assay. Then the fold change was calculated using delta Ct (2^-ΔΔCt) method.

The datasets generated during and/or analysed during the current study are available from the corresponding author on reasonable request.
